# The impact of p53 on the early stage replication of retrovirus

**DOI:** 10.1186/s12985-017-0820-7

**Published:** 2017-08-09

**Authors:** Michaela Kinnetz, Faris Alghamdi, Michael Racz, Wenwei Hu, Binshan Shi

**Affiliations:** 1 Department of Basic and Clinical Sciences, Albany College of Pharmacy and Health Sciences, 106 New Scotland Ave, Albany, NY 12208 USA; 2 Rutgers Cancer Institute of New Jersey, Rutgers the State University of New Jersey, New Brunswick, NJ 08903 USA

**Keywords:** p53, Cell cycle, Retrovirus, Reverse transcription, Mutation, LTR cycles, p21^Cip1^

## Abstract

**Background:**

The function of p53 in cancer biology has been studied extensively, but its role in anti-retrovirus infection has been elusive for many years. The restriction of retrovirus early stage replication by p53 was investigated in this study.

**Method:**

VSV-G pseudotyped retrovirus with GFP reporter gene was used to infect both HCT116 p53^+/+^ cells and its isogenic p53 knockout HCT116 p53^−/−^ cells. The infection was detected by flow cytometry. Reverse transcription products were quantified by real time PCR. Mutation analysis was performed after 1-LTR cycle and 2-LTR cycle DNA were amplified and PCR products were sequenced. Transcription and translation of cyclin-dependent kinase inhibitor 1 (p21^Cip1^) and SAM domain and HD domain-containing protein 1 (SAMHD1) were analyzed by TaqMan PCR and Western blot experiments. siRNA experiment was applied to study the role of p53 downstream gene p21^Cip1^ in the restriction of retrovirus infection.

**Results:**

It was found that the block of retrovirus infection in non-cycling cells was significantly attenuated in HCT116 p53^−/−^ cells when compared to HCT116 p53^+/+^ cells. It was found that both late reverse transcription products and viral 2-LTR cycle DNA were significantly increased in infected non-cycling HCT116 p53^−/−^ cells. Furthermore, the mutation frequency detected in 1-LTR DNA from HCT116 p53^+/+^ cells were significantly decreased in comparison to HCT116 p53^−/−^ cells. A higher number of insertion and deletion mutations were detected in the joint region of 2-LTR cycle DNA in infected p53^+/+^ cells. Cell cycle analysis showed retrovirus infection promoted host cell replication. Higher levels of mRNA and protein of p21^Cip1^ were found in HCT116 p53^+/+^ cells in comparison to the HCT116 p53^−/−^ cells. Furthermore, knockdown of p21^Cip1^ in non-cycling HCT116 p53^+/+^ cells significantly increased the infection.

**Conclusions:**

The results of this study showed that p53 is an important restriction factor that interferes with retrovirus infection in its early stage of replication. Our results suggested that p53 mediates the inhibition of retrovirus infection in non-cycling cells through it downstream gene p21^Cip1^, and p53 also functions to influence formation of 1-LTR cycle and 2-LTR cycle DNA.

## Background

p53 is a well-known tumor suppressor gene that plays fundamental roles in maintaining host genome fidelity [1, 2]. The function of p53 in cancer pathogenesis has been well-illustrated [3, 4], and previous studies have also showed that p53 acts as an important host factor that interferes various virus infections [5]. p53 was found in the interaction with viral proteins from a variety of DNA viruses, such as large T antigen of simian virus 40 [6, 7], E6 of human papillomavirus [8, 9], and E1b of adenovirus [10], HBx of human hepatitis B virus and LMP1 of Epstein-Barr virus [11–13]. Moreover, p53 is activated by phosphorylation after host cells are infected by viruses including vesicular stomatitis virus (VSV), newcastle disease virus (NDV), herpes simplex virus (HSV) and HIV [14, 15]. Host cell cycle status, activation of the DNA repair pathway and induction of apoptosis, which are regulated by p53, are also essential for viruses to create an environment for their replication. These viral proteins engage p53 in a way to increase infection by impacting p53 function directly or indirectly.

p53 has been found to be involved retrovirus infections, but its role has been elusive for many years. Like many other viruses, the retrovirus is a parasite, its efficient replication in target cells relies on its ability to overcome host defense mechanisms and to use cellular resources to finish its life cycle. Previous research had showed that p53 interferes with HIV-1 infection in the late stage of replication. p53 binds to HIV-1 LTR promoter and represses its transcription from integrated provirus [15–18]. However, the recognized functions of p53 also highly suggest its participation in the early stage of retrovirus replication, which starts from viral-host binding and entry, reverse transcription, cDNA transportation to nucleus, through integration into the host genome. First, retrovirus infection is highly dependent on host cell cycle status [19, 20] and p53 regulates the cell cycle. Second, the presence of retrovirus RNA genome, the RNA-DNA heteroduplex, and linear cDNA produced during reverse transcription all have the potential to trigger DNA damage signals, which activate the host DNA repair pathway, while p53 is the main regulator in cellular response to DNA damage. Furthermore, the generation of episomal forms of viral DNA containing either one long-terminal repeat (1-LTR circle) or two long-terminal repeats (2-LTR circle) is dependent on host cell’s DNA double-strand break repair pathways. Retrovirus 2-LTR circles are made by the non-homologous DNA end-joining (NHEJ) pathway and 1-LTR circles are produced by homologous recombination [21, 22]. p53 is involved in the regulation of homologous recombination [22]. It has been suggested that the completion of retrovirus integration also requires the participation of unidentified host enzymes [23]. p53 was found to interact with HIV reverse transcriptase by enhancing its accuracy of DNA synthesis with its 3′ to 5′ exonuclease activity [24]. Studying the role of p53 in retrovirus infection is necessary for both using retrovirus vector as a tool in gene therapy and understanding the molecular mechanism between viral host interactions in the course of infection.

In this study, human colon cancer p53 knockout cells HCT116 p53^−/−^ and its isogenic p53 wild type HCT116 p53^+/+^ cells are used to investigate the roles of p53 in early replication of retrovirus.

## Methods

### Cell culture

Human colon cancer HCT116 p53^+/+^ cells, HCT116 p53^−/−^ cells, and retrovirus packaging cell line GP2-293 (Clontech, Mountain View, CA, USA) were maintained in Dulbecco’s modified Eagle’s medium (DMEM) (Life Technologies, Grand Island, NY, USA) supplemented with 10% fetal bovine serum (FBS), 2 mM L-Glutamine, 100 units/ml of penicillin and 100 μg/ml of streptomycin at 37 °C with 5% CO_2_. In the preparation of non-cycling cells 2 × 10^6^ cells were cultured in a 10 cm dish. After 24 h cells were washed with PBS and cultured in DMEM medium without 0.5% fetal bovine serum (FBS) for 48 h. Cell viability was determined by a trypan blue exclusion assay. Cells were stained with 0.4% solution of trypan blue (Life Technologies, Grand Island, NY, USA) in phosphate buffered saline (PBS), and a hemocytometer was used to count the number of blue stained cells and total cells.

### Production of VSV-G pseudotyped retrovirus

To produce VSV-G pseudotyped retrovirus, 4 × 10^6^ of GP2-﻿﻿293 cells (Clontech Laboratories, Inc., Mountain View, CA, USA) were cultured in a 10 cm dish for 24 h, then co-transfected with plasmids pRetroX-IRES-ZsGreen1 (Clontech Laboratories, Inc., Mountain View, CA, USA) and pVSV-G (Clontech Laboratories, Inc., Mountain View, CA, USA) by using X-tremeGENE 9 DNA Transfection Reagent (Roche, Indianapolis, IN, USA.). Cells were washed with PBS after 24 h and the virus-containing culture supernatant was harvested after 48 h. After centrifugation to remove residual cells, the virus containing supernatant was aliquoted and kept in an −80 °C freezer.

### Infection

A 24 well plate was coated with 0.5 ml 20 μg/ml RetroNectin (TaKaRa, Mountain View, CA, USA) by following the manufacturer’s instructions. Each RetroNectin coated well was incubated with 0.5 ml of retroviruses for 6 h at 37 °C to allow the retrovirus to bind to RetroNectin, then unbound viruses were removed. After being washed with PBS, each well was added with 2.5 × 10^5^ HCT116 cells for infection. The infected cells were harvested at various time points post infection as designed by the experiment. In SYBR Green real time PCR experiments, the virus was treated with 4 units/ml Turbo DNase (Life Technologies, Grand Island, NY, USA) at 37 °C for 1 h to remove plasmid carry over before the infection. A heat inactivated virus control was made by incubation at 65 °C for 1 h and used in a parallel infection experiment to confirm the removal of remaining plasmid.

### Flow cytometry

Flow cytometry was used for both cell cycle analysis and quantification of infection. For cell cycle analysis, cells were washed with PBS, fixed with ice-cold 70% ethanol, and stained with 0.1% (*v*/v) Triton X-100, 20 μg/ml propidium iodide (PI) (Sigma, St. Louis, MO, USA) and 100 μg/ml DNase-free RNase (Life Technologies, Grand Island, NY, USA). For the infection assay, cells were disassociated by trypsin and washed with PBS. The infected GFP^+^ cells and uninfected cells were analyzed and quantified by a BD FACSVerse™ flow cytometer (BD Biosciences, San Jose, CA, USA). The FACSuite (BD Biosciences, San Jose, CA, USA) and the FlowJo (Ashland, OR) software were used for data analysis.

### Real time PCR

All PCR primers are listed in Table 1. For retrovirus RNA quantification, viral RNA was extracted from transfection supernatant by using RNeasy plus Mini Kit (Qiagen, Hilden, Germany). The RNA was treated with 5 units of RNase free DNase (Roche, Mannheim, Germany) to remove any remaining plasmid. Reverse transcription was performed using SuperScript® III First-Strand Synthesis Kit (Life Technologies, Grand Island, NY, USA). A series of 10-fold dilutions from 10^6^ to 10^2^ copies of template pRetroX-IRES-ZsGreen 1 was used as standards. A standard curve method was used to determine the copy number of viral RNA. For the quantification of late reverse transcription (RT) products and 2-LTR cycle DNA, DNA was extracted from infected cells by using DNeasy Blood & Tissue Kit (Qiagen, Hilden, Germany). The relative amount of late RT product and 2 LTR cycle DNA was detected and normalized to reference gene GAPDH using the real time PCR ΔΔCt method. Power SYBR® Green master mix (Life Technologies, Grand Island, NY, USA) was used for both viral RNA and DNA quantification experiments. Melting curve analysis was also performed to monitor and confirm the target gene amplification. SAMHD1, p21^Cip1^ and reference gene GAPDH TaqMan real time assays were purchased from Life Technologies. Real time PCR reactions were carried out in a StepOne Plus real time PCR instrument (Life Technologies, Grand Island, NY, USA). StepOne software was used for quantitative analysis.

**Table 1:** List of PCR primers

Name of primers	5′ to 3’	Target
RT-F	ACTTATCTGTGTCTGTCCGATTG	Late RT
RT-R	GAACTCGTCAGTTCCACCAC	Late RT
LTR-F	CTGAGCGGCTTCGAGGATAAA	1-LTR and 2-LTR
LTR-R	ACGCAGGCGCATAAAATCAG	1-LTR and 2-LTR
2-LTR-F	TGTGGTCTCGCTGTTCCTTG	2-LTR
2-LTR-R	AGTTGCATCCGACTTGTGGT	2-LTR
β-globin-F	CCCTTGGACCCAGAGGTTCT	β-globin
β-globin-R	CGAGCACTTTCTTGCCATGA	β-globin

### Viral LTR sequence analysis

The primer LTR-F is located before the 3′ LTR, and the LTR-R is located after the 5′ LTR. Therefore, both the complete length of viral 1-LTR and 2-LTR can be amplified by PCR using primer LTR-F and LTR-R. Platinum Taq DNA Polymerase (Life Technologies, Grand Island, NY, USA) was used. After agarose gel electrophoresis 1-LTR cycle and 2-LTR cycle DNA were purified by Qiagen QIAquick PCR purification kit (Qiagen, Hilden, Germany), and cloned by using TOPO® TA Cloning® Kits for sequencing (Life Technologies, Grand Island, NY, USA). DNA sequencing was performed using ABI Prism 3730XL capillary-based DNA sequencer. BioEdit computer software was used for sequence alignment [25]. The web based tool Highlight at Los Alamos HIV sequence database (http://www.hiv.lanl.gov) was used for mutation analysis of LTR sequences [26].

### Western blot

Proteins from cells lysed with Laemmli buffer (BioRad, Hercules, CA, USA) were resolved by SDS-PAGE, transferred onto PVDF membrane (Millipore, Billerica, MA) and probed with the indicated antibodies. The primary antibodies used were: anti-p21^Cip1^ (#2947) and anti-anti-phospho-SAMHD1 (Thr592) (#89930) (Cell Signaling Technologies, Danvers, MA), anti-SAMHD1 (#12586-1-AP) and anti-GAPDH (#60004-1-Ig) (Proteintech Group, Inc. Rosemont, IL), anti-p53 (#sc-126, Santa Crus, Dallas, TX), and anti-β-actin (#A5441, Sigma-Aldrich, St. Louis, MO). SuperSignal™ West Pico Chemiluminescent Substrate (Thermo Fisher, Rockford, lL, USA) was used for signal development. Chemiluminescent blot imaging was done with ChemiDoc™ Imaging Systems (BioRad, Hercules, CA, USA).

### siRNA transfection

Interfering RNA (siRNA) p21^Cip1^ (#4390824) and negative control non-target siRNA (#4392420) were purchased from Life Technologies (Life Technologies, Grand Island, NY). siRNA transfections were performed using Lipofectamine® RNAiMAX™ Transfection Reagent (Life Technologies, Grand Island, NY) according to the manufacturer’s instructions. After transfected with siRNA for 2 days, cells were cultured in DMEM with 0.5% FBS for another 24 h before infection. siRNA knockdown was confirmed by Western Blot, and infection was quantified by flow cytometer.

### Statistical tests

The Student’s t-test was used to evaluate the difference in the amount of total RT, 2-LTR, and the gene expression levels of SAMHD1 and p21^Cip1^ between infected HCT116 p53^+/+^ cells and HCT116 p53^−/−^ cells by real time PCR. Fisher’s exact test was used to evaluate the difference in the numbers of LTR clones that carry higher numbers of mutations between infected HCT116 p53^+/+^ cells and HCT116 p53^−/−^ cells. *P*-values between 0.01 and 0.05, and less than 0.01 were considered significant and highly significant, respectively.

## Results

### The block of retrovirus infection in non-cycling cells was attenuated in HCT116 p53^−/−^ cells

**Fig. 1:**
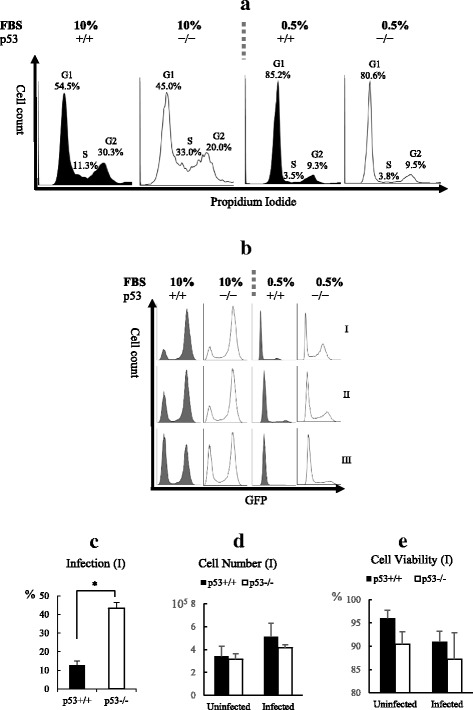
Analysis of Retrovirus Infection in Cycling and Non-Cycling HCT p53^+/+^ and HCT116 p53^−/−^ Cells. **a**. Flow cytometer analysis of cell cycle of cycling and non-cycling HCT p53^+/+^ and HCT116 p53^−/−^ cells. Non-cycling cells were cultured in DMEM with 0.5% FBS for 48 h. Cells are stained with propidium iodide (PI). **b**. Flow cytometer analysis of retrovirus infection in cycling HCT p53^+/+^ and HCT116 p53^−/−^ cells. Uninfected cells were shown as the 1st peak, and the infected GFP^+^ cells were shown as the 2nd peak. 0.5 ml of 5 × 10^7^ (I), 2.5 × 10^7^ (II) and 1.25 × 10^7^ (III) copies/ml of VSV-G pseudotyped retrovirus were used to infect 2.5 × 10^5^ cells in each well in a 24 well plate. **c**. The percentage of GFP+ positive cells were quantified by a flow cytometer after infection of non-cycling HCT p53^+/+^ and HCT116 p53^−/−^ by 0.5 ml of 5 × 10^7^ (I) copies/ml of VSV-G pseudotyped retrovirus, and the cell numbers (**d**) and viabilities (**e**) of the uninfected and infected cells were determined. The results represented a triplicate experiment. In Student’s t-test, *p* value < 0.05 is indicated by *

The impact of p53 on replication of the retrovirus was analyzed in both cycling and non-cycling HCT116 p53^+/+^ cells and HCT116 p53^−/−^ cells. The cell cycle status was analyzed by flow cytometer after PI staining. When cultured in DMEM with 10% FBS, cycling HCT116 p53^+/+^ cells and HCT116 p53^−/−^ cells had a large proportion of S phase and G2 phase cells (Fig. 1a). Non-cycling cells were prepared by culturing with serum starvation. After being cultured in DMEM with 0.5% FBS for 48 h the majority of cell populations were in G_1_ phase. Very low percentages of the cell population were in S phase in both HCT116 p53^+/+^ cells (3.5%) and HCT116 p53^−/−^ cells (3.8%), as indicated by flow cytometer analysis after PI staining (Fig. 1a).

The retrovirus was used to infect both cycling and non-cycling HCT116 cells. The VSV-G pseudotyped retrovirus carries a ZsGreen1 GFP reporter, so the infected cells were GFP positive. Cycling HCT116 p53^+/+^cells and cycling HCT116 p53^−/−^ were equally susceptible to retrovirus infection, and the infection percentages were dependent on the dosage of the virus (Fig. 1b, left panel). In the non-cycling status, HCT116 p53^+/+^ cells were highly impermeable to retrovirus infection, however the block of retrovirus infection in non-cycling HCT116 p53^−/−^ cells were significantly attenuated (Fig. 1b, right panel). There was a dosage dependent increase in the infection of non-cycling HCT116 p53^−/−^ cells. The difference in retrovirus infection between non-cycling HCT116 p53^+/+^ cells and non-cycling HCT116 p53−/− cells was further analyzed. 2.5 × 10^5^ of non-cycling HCT116 p53^+/+^ cells and non-cycling HCT116 p53^−/−^ cells were infected with 0.5 ml of 2.5 × 10^7^ copies/ml retrovirus (I). At 48 h post infection the percentage of GFP^+^ cells in non-cycling HCT116 p53^+/+^ cells (12.8 ± 2.3%) was significantly lower than the percentage of GFP^+^ cells in non-cycling HCT116 p53^−/−^ (43.4 ± 3.0%) (Fig. 1c). There was no difference in the cell number and viability between non-cycling HCT116 p53^+/+^ and HCT116 p53^−/−^ cells at 48 h post infection (Fig. 1d, e).

### The replication of retrovirus was blocked at the stage of reverse transcription in non-cycling HCT116 p53^+/+^ cells

**Fig. 2:**
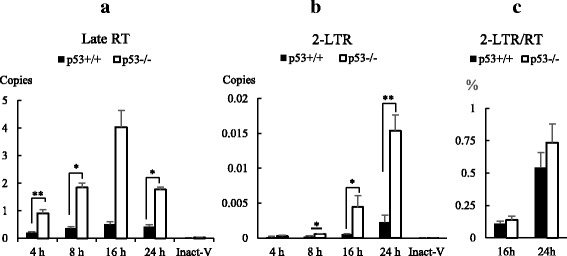
Quantification of Late RT and 2-LTR Cycle Viral DNA in Retrovirus Infected Non-Cycling HCT 116 p53^+/+^ and HCT116 p53^−/−^ Cells. 2.5 × 10^5^ cells of non-cycling HCT 116 p53^+/+^ and HCT116 p53^−/−^ cells were infected with 0.5 ml 5 × 10^7^ copies/ml retrovirus. The DNA were extracted in infected cells at 4 h, 8 h, 16 h and 24 h post infection. A heat inactivated virus (Inact-V) was used as negative control. The amount of viral late RT (**a**) and 2-LTR (**b**) in extracted DNA were quantified by SRBR Green real time PCR. The ratios of the amount between 2-LTR and RT (2LTR/RT) in infected cells were also calculated (**c**). The results represented a triplicate experiment. In Student’s t-test, *p* value < 0.05 is indicated by *; *p* value < 0.01 is indicated by **

After 2.5 × 10^5^ of non-cycling HCT116 p53^+/+^ cells and non-cycling HCT116 p53^−/−^ cells were infected with 0.5 ml of 2.5 × 10^7^ copies/ml retrovirus, cellular DNA were extracted for real time PCR analysis. The result showed that the amount of late RT product in non-cycling HCT116 p53^+/+^ cells was significantly decreased at time points of 4 h, 8 h, and 24 h after infection in comparison to infected non-cycling HCT116 p53^−/−^ cells (Fig. 2a). Real time PCR also showed the amount of 2-LTR in non-cycling HCT116 p53^+/+^ cells were significantly decreased at 8 h, 16 h and 24 h after infection (Fig. 2b). 2-LTR cycle DNA are formed after linear RT products are transported into the cell nucleus. However, the ratios between total RT products and 2-LTR cycle DNA (2-LTR/RT) did not show difference between infected non-cycling HCT116 p53^+/+^ cells and non-cycling HCT116 p53^−/−^ cells (Fig. 2c). This data suggested that the block of retrovirus infection in non-cycling HCT116 p53^+/+^ cells occurred at the reverse transcription stage by a process dependent on p53.

### Sequence analysis of retrovirus 1-LTR cycle and 2-LTR cycle DNA in infected non-cycling HCT116 p53^+/+^ cells and HCT116 p53^−/−^ cells

**Fig. 3:**
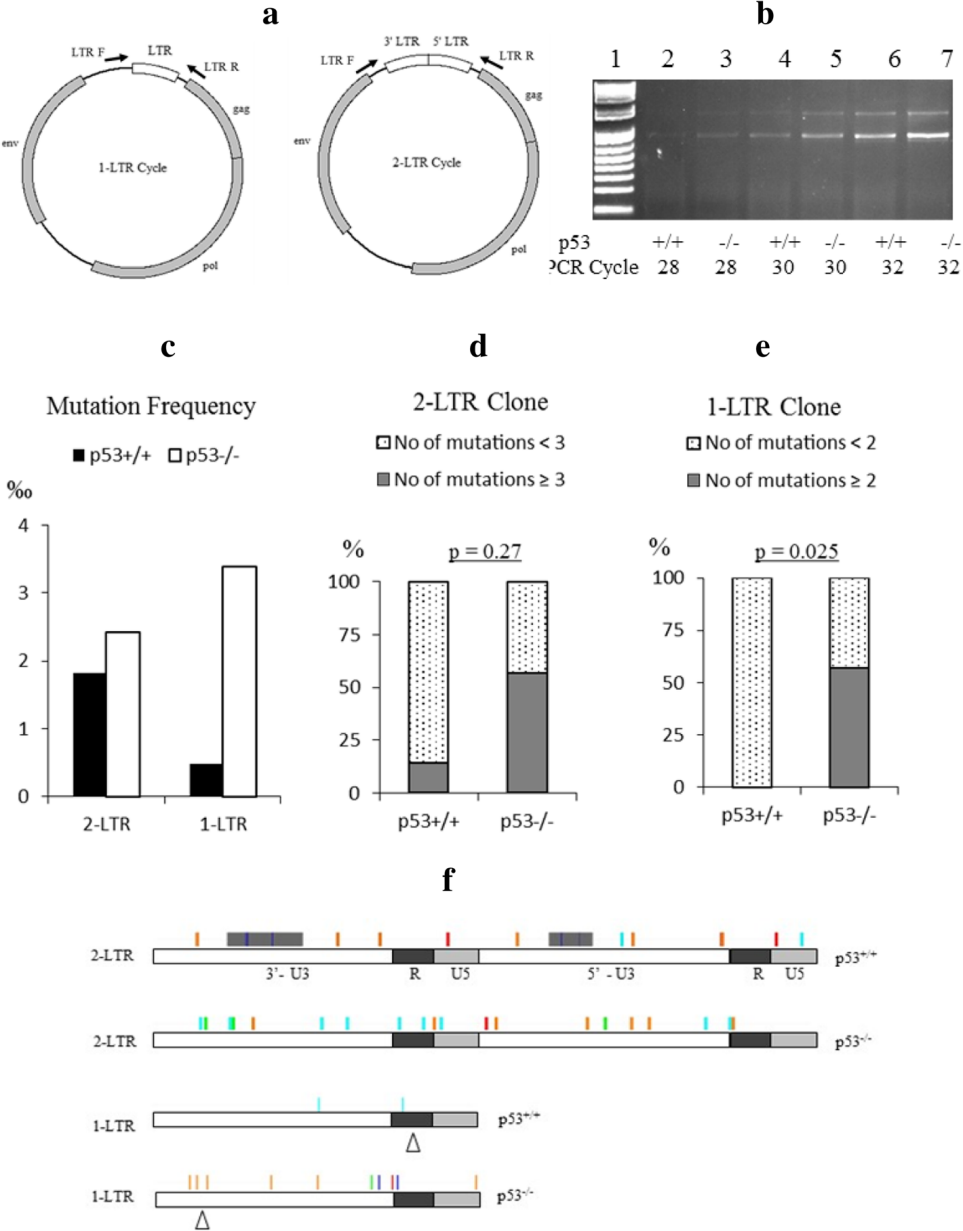
Sequence Analysis of Retrovirus 1-LTR and 2-LTR DNA in Infected Non-Cycling HCT116 p53^+/+^ and HCT116 p53^−/−^ Cells. **a**: Design of LTR PCR with primer locations. **b**. Agarose gel electrophoresis of LTR DNA PCR products. The upper bands were 2-LTR cycle DNA, and the lower bands were 1- LTR cycle DNA. Both 1-LTR and 2-LTR cycle DNA were cloned and then sequenced. **c**: The overall mutation frequency detected in all clones of 1-LTR and 2-LTR cycle DNA from infected HCT116 p53^+/+^ and HCT116 p53^−/−^ cells. **d**. The comparison of number of mutations in the 2-LTR cycle DNA clones between infected non-cycling HCT116 p53^+/+^ and HCT116 p53^−/−^ cells. **e**. The comparison of number of mutations in the 1-LTR cycle DNA clones between infected non-cycling HCT116 p53^+/+^ and HCT116 p53^−/−^ cells. The *p* value of Fisher’s test is shown above the chart. **f**. The distribution of mutations in 1-LTR and 2-LTR DNA in infected non-cycling HCT116 p53^+/+^ and HCT116 p53^−/−^ cells. Mismatches are shown with color bars. Green represents mutant base A; Red represents base mutant T; Orange represents mutant base G; Light blue represents mutant base C; Gray represent gaps and Δ represents insertions

A PCR was designed so that both 1-LTR cycle and 2-LTR cycle DNA can be amplified from infected cells simultaneously (Fig. 3a). The amplified 1-LTR DNA had a band of 785 bp and the 2-LTR DNA had a band of 1377 bp. The 1-LTR bands had higher intensity than the 2-LTR bands (Fig. 3b). The 1-LTR and 2-LTR DNA amplified in the early PCR cycle was gel purified and cloned into TOPO-PCR vector. Sequencing of cloned 1-LTR and 2-LTR DNA was performed. Sequences from seven 2-LTR cycle clones (total 9.6 kb) from infected non-cycling HCT116 p53^+/+^ and seven 2-LTR cycle clones (total 9.6 kb) from non-cycling HCT116 p53^−/−^ cells were analyzed. The retrovirus 2-LTR cycle DNA isolated from infected non-cycling HCT116 p53^+/+^ and non-cycling HCT116 p53^−/−^ cells had mutation frequencies of 1.81‰ and 2.41‰ respectively. Eight 1-LTR cycle clones each from infected non-cycling HCT116 p53^+/+^ and non-cycling HCT116 p53^−/−^ cells were sequenced (total 6.2 kb from each cell lines). The retrovirus 1-LTR cycle DNA isolated from infected non-cycling HCT116 p53^+/+^ and non-cycling HCT116 p53^−/−^ cells had mutation frequencies of 0.48‰ and 3.38‰ respectively (Fig. 3c). More 2-LTR clones from infected non-cycling HCT116 p53^+/+^ contained a lower number of mutations (86% of clones had less than 3 mutations) than 2-LTR clones from infected non-cycling HCT116 p53^−/−^ cells (43% of clones had more than 3 mutations) (Fig. 3d). No 1-LTR clones from infected non-cycling HCT116 p53^+/+^contained more than 2 mutations, while 57% of 1-LTR clones from non-cycling HCT116 p53^−/−^ cells contained more than 2 mutations (*p* = 0.025) (Fig. 3e).

Mutations detected in 1-LTR cycle and 2-LTR cycle DNA in infected non-cycling HCT116 p53^+/+^ cells and HCT116 p53^−/−^ cells were mostly randomly distributed (Fig. 3f). There were no mutation hotspots identified. Two out of seven clones (29%) of 2-LTR from HCT116 p53^+/+^ cells contained 72 bp deletions in different locations in U3 region of LTR, but no deletions were found in 2-LTR clones from p53^−/−^ cells, indicating an increased deletion in the 2-LTR region of HCT116 p53^+/+^cells compared to HCT116 p53^−/−^ cells. One 1-LTR clone isolated from p53^+/+^ contained a 31 bp insertion in the R region, and one 1-LTR clone from p53^−/−^ cells had a 75 bp insertion in the U3 region (Fig. 3f).

### Sequence analysis of joint region of 2-LTR cycle DNA from infected non-cycling HCT116 p53^+/+^ cells and HCT116 p53^−/−^ cells

The sequence analysis of the joint region of 2-LTR cycle DNA may reveal the function of viral RNase H and other cellular nucleases that might be involved in the processing of viral cDNA free ends. The 2-LTR joint region sequences from seven clones of infected non-cycling HCT116 p53^+/+^ cells and seven clones from HCT116 p53^−/−^ cells were aligned and are shown in Table 2. Four out of seven clones (57%) of 2-LTR from infected non-cycling HCT116 p53^+/+^ cells contained 1-4 bp insertions or deletions, while only one out of seven (14%) 2-LTR clones from infected non-cycling HCT116 p53^−/−^ cells contained a 4 bp insertion. This result indicated an increased number of insertions and deletions were found in the joint region of 2-LTR DNA in infected p53^+/+^ cells.

**Table 2:**
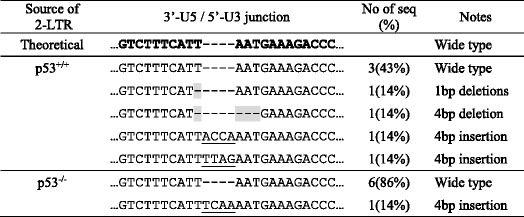
Sequence alignment of 2-LTR joint region from clones isolated from infected non-cycling HCT116 p53^+/+^ cells and HCT116 p53^−/−^ cells

All sequences containing a deletion or insertion are aligned to the wide type sequence. Deleted sequences are highlighted in gray. Inserted sequences are underlined. 2-LTR clones were isolated from cells 24 h after infection by retrovirus

### Cell cycle status change post retrovirus infection

**Fig. 4:**
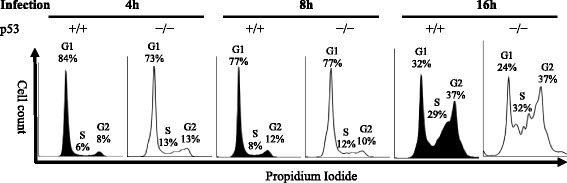
Cell Cycle Change after Retrovirus Infection. 2.5 × 10^5^ cells of non-cycling HCT 116 p53^+/+^ and HCT116 p53^−/−^ cells were infected with 0.5 ml 2.5 × 10^7^ copies/ml retrovirus. After 4 h, 8 h and 16 h cells were harvested, stained by PI and analyzed by flow cytometer

In the above experiments, HCT116 p53^+/+^ cells and HCT116 p53^−/−^ cells were cultured in DMEM with 0.5% FBS before and after infection. Since cells were subcultured and transferred to RetroNectin coated virus bound wells and retrovirus infection may also induce host cell cycle condition change after infection, cell cycle statuses of infected HCT116 p53^+/+^ cells and HCT116 p53^−/−^ cells were analyzed by flow cytometer post infection. There was no noticeable increase of S and G_2_ phase cells in both HCT116 p53^+/+^ and HCT116 p53^−/−^ cells 4 h and 8 h post infection, but significant increases in S and G_2_ phase cells were found in both HCT116 p53^+/+^ cells and HCT116 p53^−/−^ cells 16 h post infection (Fig. 4). This result indicated retrovirus infection may promote host cell cycle progression.

### Analysis of SAMHD1 and p21^Cip1^ gene expression in HCT116 p53^+/+^ and p53^−/−^ cells

**Fig. 5:**
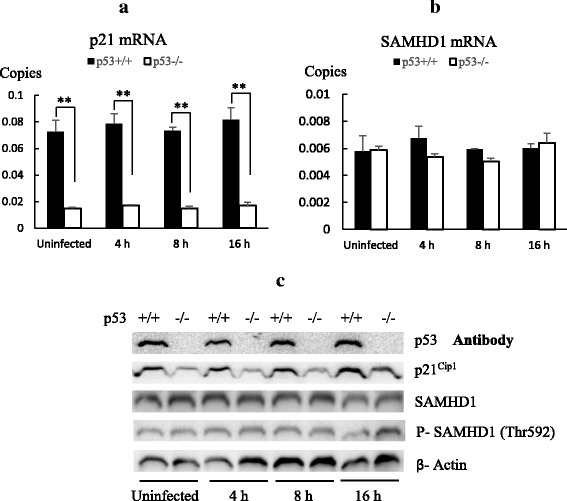
Analysis of SAMHD1 and p21^Cip1^ gene expression in HCTp53^+/+^ and HCT116 p53^−/−^ cells. 2.5 × 10^5^ cells of non-cycling HCT 116 p53^+/+^ and HCT116 p53^−/−^ cells cultured in DMEM with 0.5% FBS (Un-infected), and were infected with 0.5 ml 2.5 × 10^7^ copies/ml retrovirus for 4 h, 8 h and 16 h. RNA were extracted from cells harvested, and then analyzed by p21^Cip1^ TaqMan real time PCR (**a**), SAMHD1 TaqMan real time PCR (**b**). The copy numbers of mRNA were normalized to reference gene GAPDH. p53, p21^Cip1^, SAMHD1, phospho-SAMHD1 (Thr592) and β-actin proteins were analyzed by Western blot (**c**) . *p*-value of Student’s t-test is indicated. *p* value < 0.01 is indicated by **

SAMHD1 and p21^Cip1^ were previously reported to block reverse transcription of HIV [27, 28]. Gene expressions of SAMHD1 and p21^Cip1^ in HCT116 p53^+/+^ and HCT116 p53^−/−^ cells were analyzed using TaqMan real time PCR and Western blot before and 4 h, 8 h and 16 h post infection. The gene expression of p21^Cip1^ was significantly higher in HCT116 p53^+/+^ than HCT116 p53^−/−^ cells at both mRNA and protein levels (Fig. 5a and c). However, SAMHD1 expression did not show difference at both transcription and translation levels between HCT116 p53^+/+^ and HCT116 p53^−/−^ cells (Fig. 5b and c). Phosphorylation of SAMHD1 at Thr 592 results in the inactivation of its antiretroviral function, but there were also no detectable difference of phosphorylated SAMHD1 between non-cycling uninfected and infected HCT116 p53^+/+^ and HCT116 p53^−/−^ cells (Fig. 5c). This data suggested p21^Cip1^ may be responsible for the block of reverse transcription in HCT116 p53^+/+^, and this block was dependent on the cell cycle status at time of infection.

### The block of retrovirus infection in non-cycling HCT116 p53^+/+^ cells was attenuated by p21^Cip1^ siRNA knockdown.

**Fig. 6:**
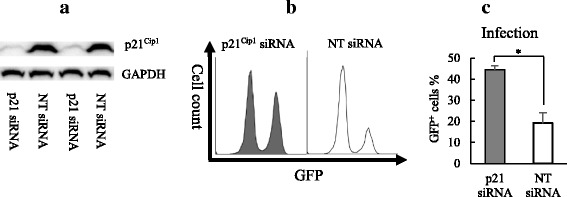
The Block of Retrovirus Infection in Non-Cycling HCT116 p53^+/+^ Cells Was Attenuated by p21^Cip1^ siRNA Knockdown. The knockdown of p21^Cip1^ by siRNA in HCT116 p53^+/+^ cells were testified by Western Blot (**a**). p21^Cip1^ siRNA transfected non-cycling HCT116 p53^+/+^ cells were infected retrovirus. The infected GFP^+^ cells were analyzed by flow cytometer (**b**), and the percentage of infections was calculated (**c**). Non-target siRNA (NT) was used as negative control. The results represented a duplicated experiment. In Student’s t-test, *p* value < 0.05 is indicated by *

To investigate whether the high level of p21^Cip1^ caused block of retrovirus infection in non-cycling HCT116 p53^+/+^ cells, knockdown of p21^Cip1^ gene expression by siRNA was carried out. The Western blot result clearly showed that the p21^Cip1^ gene expression was decreased after siRNA transfection (Fig. 6a). Two days after siRNA transfection HCT116 p53^+/+^ cells were cultured with DMEM with 0.5% FBS for 24 h, and then were infected with retrovirus. It was found that the infection was significantly increased in p21^Cip1^ siRNA treated HCT HCT116 p53^+/+^ cells in comparison to non-target siRNA treated cells by using flow cytometer analysis (Fig. 6b and c), indicating that p21^Cip1^ was responsible for the observed block of retrovirus infection in non-cycling cells.

## Discussion

The retrovirus vector based virus has been commonly applied in virology studies and in gene delivery experiments [29]. In a single round of infection, VSV-G pseudotyped retrovirus goes through essential steps in retrovirus replication including entry, reverse transcription, viral cDNA nuclear transportation, integration and expression. This infection experimental system provides an ideal model for us to study the role of p53 in early replication of retrovirus.

The use of serum depletion in cell culture was previously reported to create cell cycle G_0_ arrest, which produces non-cycling cells [19]. Our experiment results by using flow cytometry analysis indicated that the majority HCT116 p53^+/+^ cells and HCT116 p53^−/−^ cells were non-cycling cells after 48 h serum depletion. The RetroNectin (Clontech) used in this study is a recombinant fibronectin fragment that was previously reported to improve transduction of retrovirus [30]. After a virus bound plate was prepared, the virus-containing supernatant was removed and washed with PBS before the addition of cells, which avoided serum in the culture medium in the non-cycling cell infection experiment. Cell division has been known to be a requirement for successful replication of retrovirus. In our study, the retrovirus infection of HCT116 p53^+/+^ was greatly inhibited after the cell cycle was stopped at G_0_, which agrees with previous conclusions by Lewis et al. and Roe et al. [19, 20]. However, the block of infection in non-cycling cells was significantly attenuated in HCT116 p53^−/−^ cells, suggesting the block of retrovirus infection in non-cycling cells could be mediated by p53. The subculture and transferring of cells to RetroNectin coated virus bound plate before infection may stimulate cells to grow, and the retrovirus may also induce the host cell cycle progression to benefit its replication. As a result, we observed cell cycle changes at 16 h post infection even when infected cells were cultured in DMEM with 0.5% FBS. About 60% of the infected cell population entered S and G_2_ phases 16 h post infection. Therefore, this result suggested the p53 regulated permeability to retrovirus infection might be highly dependent on the cell cycle status at the time of infection.

In an early study, Harel showed that the amount of unintegrated linear viral DNA was decreased in MLV infected quiescent NIH/3 T3 cells after serum depletion [31]. Both Zack et al. and Kootstra et al. demonstrated that HIV-1 reverse transcription was blocked in quiescent macrophages and lymphocytes [32, 33]. In this study we found that the block in the reverse transcription of retrovirus infection was attenuated in non-cycling HCT116 p53^−/−^ cells compared to p53^+/+^ cells, implying that a process mediated by p53 triggered the restriction at reverse transcription. The depletion of dNTPs pool by SAMHD1 in non-cycling cells had been considered as host restriction to block HIV-1 reverse transcription [28, 34]. p21^Cip1^ was also identified to inhibit HIV-1 reverse transcription independent of SAMHD1 [27, 35]. p21^Cip1^ is a well-known p53 downstream gene. Our siRNA knockdown experiment strongly indicated that p21^Cip1^ was responsible for the observed inhibition of retrovirus reverse transcription in the infection of HCT116 p53^+/+^cells. The inhibition also highly depends on the host cell cycle status at the beginning of infection.

After reverse transcription the linear viral cDNA is transported into the cell nucleus, where part of viral cDNA is autointegrated as circular forms 1-LTR cycle and 2-LTR cycle DNA. 1-LTR cycle and 2-LTR cycle DNA are formed by the action of host proteins in DNA double-strand break repair pathways. 1-LTR is formed through homologous recombination end joining DNA double-strand break repair pathway, and 2-LTR is the product of non-homologous end joining DNA double-strand break repair pathway [21, 36]. Since 2-LTR DNA is detected exclusively in the cell nucleus, it is considered a useful marker of viral nuclear import [36]. In this study the amount of 2-LTR was significantly decreased in infected non-cycling HCT116 p53^+/+^ cells compared to HCT116 p53^−/−^ cells. However ratios of the amount of 2-LTR to late reverse transcription products between HCT116 p53^+/+^ and HCT116 p53^−/−^ cells did not show a significant difference. This suggests the observed decrease in 2-LTR DNA in non-cycling HCT116 p53^+/+^ cells could be a consequence of the major block in reverse transcription, and there was no block in the nuclear transportation and formation of 2-LTR cycle DNA in non-cycling HCT116 p53^+/+^ cells.

Sequence analysis of 1-LTR and 2-LTR may reveal the influence of host factors in the process of reverse transcription and the formation of LTR. The mutations detected in 2-LTR clones may directly reflect errors in the synthesis of viral cDNA by reverse transcriptase. Bakhanashvili et al. found that p53 has 3′ to 5′ exonuclease activity and may enhance the accuracy of DNA synthesis by HIV reverse transcriptase in the cytoplasm [24]. We found the mutation frequency was decreased slightly in the 2-LTR clones in retrovirus infected HCT116 p53^+/+^ cells compared to HCT116 p53^−/−^ cells. The mutations detected in 1-LTR clones may reflect both the errors in the synthesis of viral cDNA and the errors during its formation by homologous recombination in nucleus. p53 is involved in the regulation of homologous recombination [22]. It was found in this study that the mutation frequency in 1-LTR clones in non-cycling HCT116 p53^+/+^ cells was significantly decreased compared to HCT116 p53^−/−^ cells, which suggests that p53 also influenced the precise process in the formation of 1-LTR by the homologous recombination.

In retrovirus reverse transcription, the removal of the tRNA primer defines the right end of the viral DNA, the generation and removal of the polypurine tract primer defines the left end of the viral DNA. Normally it is the activity of RNase H of reverse transcriptase that removes either the tRNA or the polypurine tract primer [37]. Our result showed that a higher frequency of insertions and deletions was detected in the joint region of 2-LTR DNA in infected HCT116 p53^+/+^ cells. This provides evidence that p53 may play a role either on the influencing the function of RNase H or on the abnormal modification of viral cDNA ends in non-cycling cells.

Infection by retrovirus was reported to activate p53 by phosphorylation [14]. The subsequent up-regulation of p21^Cip1^ induced by p53 results in the block of retrovirus reverse transcription, which may reflect one of the antiretroviral actions mediated by p53. In this study the result that inhibition of reverse transcription was mediated by p53 in non-cycling cells was based on experiments with VSV-G pseudotyped retrovirus. It will be very interesting to investigate other retrovirus such as HIV-1, whose main host cells are non-cycling and differentiated macrophages and lymphocytes. Further understanding of molecular mechanism mediated by host cell p53 in the inhibition of replication of the retrovirus in non-cycling cells will have important implications not only for the basic biology of retroviruses, but also for our understanding of viral pathogenesis.

## Conclusions

Retrovirus infection is usually blocked in non-cycling cells, however in this study it was found that this block was attenuated in p53 knockout cell HCT116 p53^−/−^. Results showed that the amount of reverse transcription and 2-LTR cycle DNA were increased in infected non-cycling HCT116 p53^−/−^cells in comparison to HCT116 p53^+/+^ cells. It was also found that mutation frequency of 1-LTR DNA was decreased in HCT116 p53^+/+^ cells, and a higher number of insertion and deletion mutations were detected in the joint region of 2-LTR DNA in infected p53^+/+^ cells in comparison to HCT116 p53^−/−^ cells. Further analysis by siRNA knockdown experiment highly suggested the increased level of p53 downstream gene p21^Cip1^ in HCT116 p53^+/+^ cells was responsible for the block of retrovirus infection in non-cycling cells. Our data has demonstrated that p53 plays important role in early replication of retrovirus.

## Abbreviations

FBS: Fetal bovine serum
HIV: The human immunodeficiency virus
LTR: Long terminal repeat sequence
p21^Cip1^: Cyclin-dependent kinase inhibitor 1.
PI: Propidium iodide
SAMHD1: SAM domain and HD domain-containing protein 1
VSV-G: Vesicular stomatitis virus G-protein
